# Ain't No Sunshine When She's Gone: Pseudohypoparathyroidism Discovered in an Adult

**DOI:** 10.1155/2012/739375

**Published:** 2012-04-03

**Authors:** C. R. van Rooijen, M. B. Kok, S. Simsek, F. Stam

**Affiliations:** ^1^Department of Internal Medicine, Medical Centre Alkmaar, Wilhelminalaan 12, 1815 JD Alkmaar, The Netherlands; ^2^Department of Clinical Chemistry, Medical Centre Alkmaar, Wilhelminalaan 12, 1815 JD Alkmaar, The Netherlands; ^3^Department of Internal Medicine, VU University Medical Centre, De Boelelaan 1117, 1007 MB Amsterdam, The Netherlands

## Abstract

An 18-year-old negroid woman presented with progressive cramps in both hands. She was Jamaican and had moved to The Netherlands 8 months before. On physical examination Trousseau's sign was positive. Laboratory analysis showed severe hypocalcaemia (1.17 mmol/L) and hyperphosphatemia (2.0 mmol/L). Urinary excretion of both calcium (0.8 mmol/day) and phosphate (5 mmol/day) was low, as is seen in hypoparathyroidism. However, the PTH level was increased (22.1 pmol/L), whereas 25-(OH)-vitamin D was low (31 nmol/L). An Ellsworth-Howard test showed only a fivefold increase in urinary phosphate excretion after administration of synthetic PTH, supporting the diagnosis pseudohypoparathyroidism. Upon treatment with calcium supplementation and alfacalcidol, her symptoms disappeared. Pseudohypoparathyroidism (PHP) is a rare hereditary disorder resembling hypoparathyroidism, although plasma PTH levels are elevated. PHP is caused by alterations in the PTH receptor, inducing target tissue resistance to PTH. This results in hypocalcaemia and hyperphosphatemia, while PTH levels are elevated. The diagnosis is confirmed by the Ellsworth-Howard test, which will show a 100-fold increase in phosphate excretion if the PTH receptor functions properly. Treatment is lifelong supplementation of calcium and alfacalcidol. In our patient, symptoms were probably evoked by the lack of sunlight in Dutch winter, decreasing vitamin D levels and thereby aggravating hypocalcaemia.

## 1. Introduction

Calcium is not only an important component of bone, it also plays a key role in generating action potentials. As a result, hypocalcaemia increases excitability of nerve and muscle cells, leading to cramps and, in severe cases, tetany. Serum calcium levels are hormonally controlled, mainly by parathyroid hormone and vitamin D. The latter is to a small extent attained from food, but the majority is formed in sun-exposed skin. The risk for deficiency is increased in dark-skinned people living at higher latitudes. We describe a case of a dark-skinned patient with extreme hypocalcaemia, caused by an unusual disorder in calcium metabolism.

## 2. Case Report

An 18-year-old negroid woman presented at the emergency department with progressive cramps in both hands for two days. She was born in Jamaica and had moved to The Netherlands eight months prior to presentation. She had no relevant medical history and did not use any medication. On physical examination, we found her fingers to be cramped with Trousseau's sign positive. No other abnormalities were observed. Laboratory analysis showed severe hypocalcaemia (1.17 mmol/L; reference value 2.10–2.50) and hyperphosphataemia (2.0 mmol/L; 0.7–1.4) with normal blood levels of albumin (36 g/L; 35–50), creatinin (61 *μ*mol/L; 50–90), and alkaline phosphatase (96 U/L; 0–120). Magnesium was slightly decreased (0.6 mmol/L; 0.7–1.0). Electrocardiography showed normal QTc time, but flattened ST segments, consistent with hypocalcaemia.

 As could be expected in a dark-skinned person in Dutch autumn, the 25OHD level was low (31 nmol/L; 50–280). However, this minor deficiency was an unlikely explanation for the severe calcium depletion. 1,25-(OH)_2_-vitamin D was low normal (69 pmol/L; 48–161). Instead, the combination of hypocalcaemia and hyperphosphataemia was considered more consistent with hypoparathyroidism, a diagnosis supported by urine analysis, showing a low excretion of both calcium (0.8 mmol/day; 2.5–7.5) and phosphate (5 mmol/day; 10–50). Unexpectedly, parathyroid hormone (PTH) was increased (22.1 pmol/L; 1.3–6.9), rendering the possibility of pseudohypoparathyroidism (PHP). To confirm this diagnosis, an Ellsworth-Howard test was performed, measuring urinary phosphate excretion after administration of a high dose of synthetic PTH. In our patient, phosphate excretion only increased fivefold, where a 100-fold increase is regarded as normal, supporting the diagnosis PHP. No other hormonal imbalances were found. Although the normal alkaline phosphatase level suggested no PTH-induced increase in bone resorption, densitometry was performed to assess bone mineral density, revealing normal values of the lumbar spine (*T* = +0.2) and hips (*T* = +1.4).

Upon treatment with calcium supplementation and 1*α*-(OH)-vitamin D (alfacalcidol), symptoms disappeared. Serum calcium levels, however, remained in the low range, as did her calcium excretion.

## 3. Discussion

Pseudohypoparathyroidism (PHP) is a rare disorder with clinical and biochemical features mimicking hypoparathyroidism, although plasma PTH levels are elevated [1]. Normally, PTH increases serum calcium levels by stimulating bone resorption and renal calcium reabsorption. Furthermore, conversion of 25OHD to the metabolic active 1,25-(OH)_2_-vitamin D (calcitriol) is stimulated. Calcitriol and PTH interact to increase intestinal calcium and phosphate absorption, whereas PTH also stimulates renal phosphate excretion [2].

In PHP the target tissue is resistant to PTH, resulting in hypocalcaemia and hyperphosphatemia. Consequently, the normal functioning parathyroid glands will further increase PTH secretion [1].

Resistance to PTH is caused by alterations of the PTH receptor, which is encoded by the GNAS1 gene [3, 4].

Different types of PHP, each with specific features, have been described (Table 1). The best known type of PHP is type 1a, where biochemical disruptions are combined with a phenotype called Albright's hereditary osteodystrophy (AHO), including short stature, round face, brachymetacarpia, and subcutaneous ossifications [1]. The origin of PTH resistance accounts for the differences between the types. The GNAS1 gene, encoding the PTH receptor, can either be mutated (PHP type 1a and pseudo-PHP) or its methylation can be altered (PHP type 1b). In most target tissues both maternal and paternal alleles are transcriptionally active. However, in the proximal tubulus only the maternal allele is read. Thus, changes in the paternal *GNAS1* allele do not lead to electrolyte imbalances [3, 4]. In our patient, who presented with biochemical abnormalities and a normal phenotype, the most likely diagnosis is PHP type 1b, a methylation defect of the maternal *GNAS1* gene. This is supported by the fact that a mutation in *GNAS1* could not be demonstrated. However, type 2 PHP, where the problem originates from the signaling cascade of PTH, cannot be ruled out.

Since all types of PHP are congenital and the patient did not experience any symptoms during the first 18 years of her life, we believe moving to The Netherlands aggravated her hypocalcaemia. Possibly, the lack of sunshine in Dutch winter resulted in decreased vitamin D levels, lowering calcium levels even further. Strangely, even with low 25OHD levels and a PTH resistant kidney, calcitriol levels were within the normal range. Adaptation might have occurred by augmenting the normal activation of 25OHD in other tissues. We assume that vitamin D levels were significantly higher when living in a sunnier climate.

In addition, the normal bone mineral density remains partly unexplained. In theory, the bones are sensitive to PTH, in PHP type 1b. Therefore, osteoporosis, caused by high circulating PTH levels, would be expected. Reality, however, is not completely consistent. In the few cases described, both osteosclerosis and osteoporosis were found [5]. Possible explanations for the differences can be incomplete penetrance in familial forms and different degrees of GNAS methylation [6].

Moreover, reference values for bone densitometry are based on investigations in Caucasian women, and uniform, non-race-adjusted T-scores are utilized [7].

## 4. Treatment

Treatment of PHP is lifelong suppletion of calcium and 1*α*-(OH)-vitamin D (alfacalcidol) under strict monitoring of serum and urinary calcium levels [2]. Upon achieving normocalcaemia, calcium excretion will increase quickly, resulting in a condition associated with a considerable risk of urinary stone formation. Unfortunately, the patient was noncompliant and stopped taking medication as soon as her complaints disappeared. With a calcium level as low as 1.32 mmol/l, she had no complaints and could not be motivated to take pills on a daily base. This illustrates how well the body adapts to prolonged hypocalcaemia.

## Figures and Tables

**Table 1:** Different types of pseudohypoparathyroidism. *Methylation defect in maternal allele, **disrupted PTH signaling cascade, AHO: Albright's hereditary osteodystrophy, PHP: pseudohypoparathyroidism.

	PHP type 1a	Pseudo PHP	PHP type 1b*	PHP type 2**	Patient
Phenotype	AHO	AHO	normal	normal	normal
Biochemical changes	+	−	+	+	+
PTH resistance in kidneys	+	−	+	+	+
PTH resistance in other tissue	+	+	−	−	−
GNAS mutation	maternal allele	paternal allele	−	−	−
